# Racial and ethnic disparities in utilization of mechanical circulatory support for cardiogenic shock following acute myocardial infarction: a retrospective cross-sectional study

**DOI:** 10.1097/MS9.0000000000004407

**Published:** 2025-11-24

**Authors:** Aimen Shafiq, Ali Salman, Ahsan Alam, Summiya Qureshi, Anas M. Din Bashir, Umair Bajwa, Bushra Ishaq, Khushal Khan, Hassan Waheed Malik, Zainab Mubasher, Fnu Laiba, Mahnoor Shah, Muhammad Talha Saghir, Osman Wafai, Asad Ali Ahmed Cheema

**Affiliations:** a Department of Medicine, Dow University of Health Sciences, Karachi, Pakistan; b Department of Medicine, Ascension Borgess Medical Center, Kalamazoo, MI, United States; c Department of Medicine, Fatima Jinnah Medical University, Karachi, Pakistan; d Department of Pharmacology, Shalamar Medical & Dental College, Lahore, Pakistan; e Department of Medicine, Services Institute of Medical Sciences, Lahore, Pakistan; f Department of Medicine, King Edward Medical University, Lahore, Pakistan; g Department of Medicine, Aga Khan University, Karachi, Pakistan; h Department of Medicine, Lahore Medical and Dental College, Lahore, Pakistan; i Department of Medicine, Rashid Latif Medical College, Lahore, Pakistan; j Department of Medicine, Akhtar Saeed Medical & Dental College, Lahore, Pakistan; k Department of Medicine, Fatima Memorial Hospital, Lahore, Pakistan; l Department of Medicine, Jinnah Medical & Dental College, Karachi, Pakistan; m International School of Medicine, International University of Kyrgyzstan, Bishkek, Kyrgyzstan

**Keywords:** access to care, acute heart failure, device, myocardial infarction, race

## Abstract

**Background:**

Mechanical circulatory support (MCS) is critical in the management of cardiogenic shock (CS) complicating acute myocardial infarction (AMI), but racial/ethnic disparities in MCS utilization remain understudied.

**Methods:**

We extracted data from the National Inpatient Sample database for the years 2018 to 2020. Patients aged ≥18 years with AMI and CS listed as primary or secondary diagnosis were identified using ICD-10-CM. The primary outcome was in-hospital mortality. Secondary outcomes included MCS utilization, hospital length of stay (LOS), total hospital charges, acute kidney injury (AKI)/hemodialysis, and sepsis. Multivariable logistic and linear regression models were used to assess associations between race/ethnicity and in-hospital outcomes.

**Results:**

Among 89 125 hospitalizations for AMI and CS, Hispanics had lower in-hospital mortality than Whites (OR 0.86; 95% CI, 0.76–0.96). The MCS use was higher among Asians or Pacific Islanders (OR 1.35; 95% CI, 1.14–1.60) and Hispanics (OR 1.15; 95% CI, 1.01–1.30) than among Whites. Hispanics had longer LOS (*β* = 1.3 days; 95% CI, 0.78–1.7), whereas Blacks had shorter stays (*β* = −0.50 days; 95% CI,−0.91 to −0.10) than Whites. The rates of AKI/hemodialysis were higher among Hispanics (OR 1.26; 95% CI, 1.12–1.42), Blacks (OR 1.59; 95% CI, 1.40–1.79), and Asians or Pacific Islanders (OR 1.39; 95% CI, 1.18–1.64) than Whites. Sepsis rate was greater among Hispanics (OR 1.18; 95% CI, 1.04–1.34). Total hospital charges were higher among Hispanics ($53 770; 95% CI, $39 307–$68 233) and Asians or Pacific Islanders ($33 737; 95% CI, $8954–$58 520), than Whites.

**Conclusion:**

Racial and ethnic disparities in MCS use and clinical outcomes persist among patients with AMI and CS.

## Graphical abstract

**Figure:**
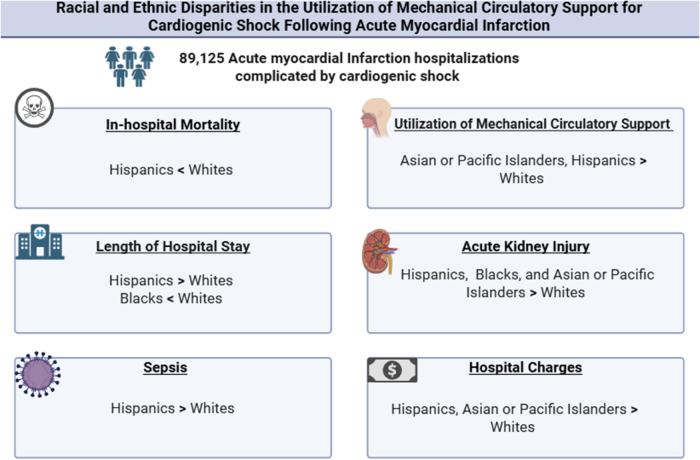

## Introduction

Cardiogenic shock (CS) is the most severe complication of acute myocardial infarction (AMI), associated with high mortality, about 3.01 per 100 000 individuals in the CS-complicating AMI population[1]. Hence, AMI-related CS requires effective and prompt management to enhance patient survival[2]. Mechanical circulatory support (MCS) devices, including intra-aortic balloon pumps (IABP), percutaneous ventricular assist devices (pVADs), and extracorporeal membrane oxygen (ECMO), have emerged as adjuncts to improve hemodynamics and outcomes in carefully selected patients with AMI and CS[3]. Although the use of MCS remains under investigation due to conflicting evidence on long-term outcomes, timely access to these interventions is critical for survival[4]. However, inequity in access and utilization of MCS remains a concern among different patient populations^[5,6]^.

Prior studies have documented disparities in the utilization of various cardiac interventions, including coronary angiography and revascularization, among different racial and ethnic groups[5]. Individuals from underrepresented populations often face reduced access to advanced therapies, delayed treatment, and worse outcomes^[6,7]^. An analysis of data from 2012 to 2017 found that Black and other racial groups had lower rates of coronary angiography, percutaneous coronary intervention (PCI), and MCS device use compared to White individuals, yet paradoxically, adjusted in-hospital mortality was lower in these groups despite differences in treatment[8]. In contrast, another study focusing on patients with ST-segment elevation myocardial infarction (STEMI) and CS reported higher in-hospital mortality among Black and Hispanic men compared to White men, highlighting inconsistencies in prior findings[9].

These mixed results underscore the need to examine further racial and ethnic differences in resource utilization and outcomes among patients with AMI and CS^[8,9]^. To address this, we analyzed data from the National Inpatient Sample (NIS) to evaluate the use of MCS across racial and ethnic groups over a 3-year period (2018–2020). By adjusting clinical and hospital-level factors, this study aims to determine whether disparities in MCS utilization persist among patients with AMI-related CS.

## Methods

### Study design and data source

We conducted a retrospective cross-sectional analysis of hospitalizations for AMI and CS using data from the NIS from 1 January 2018 to 31 December 2020. The NIS is a component of the Healthcare Cost and Utilization Project (HCUP), sponsored by the Agency for Healthcare Research and Quality (AHRQ). It is the largest publicly available all-payer inpatient healthcare database in the United States. It captures approximately 20% of all US hospital discharges from non-federal acute care hospitals and includes over seven million unweighted hospitalizations annually. When weighted, the NIS produces national estimates approximating 35 million discharges per year. The database consists of both patient-level and hospital-level data. It allows for up to 40 discharge diagnoses and 25 procedures per hospitalization, recorded using the International Classification of Diseases, 10th Revision (ICD-10) coding system. Because the NIS contains de-identified data, this study was exempt from institutional review board approval. This retrospective cross-sectional study has been designed and reported in accordance with the Revised Strengthening the Reporting of Cohort, Cross-sectional, and Case-control Studies in Surgery (STROCSS) 2025 guidelines, which aim to enhance the transparency, completeness, and reproducibility of observational research[10].

### Study population

We included adults aged 18 years or older who were hospitalized with a primary or secondary diagnosis of AMI and CS during the study period. AMI was identified using ICD-10-CM codes I21.0 through I21.4; cases coded as I21.9 were excluded to avoid the inclusion of unspecified myocardial infarctions. CS was identified using the ICD-10-CM code R57.0. To ensure complete capture of clinical outcomes during the index hospitalization, we excluded patients who were transferred into or out of another acute care facility. Hospitalizations with missing data on race/ethnicity or AMI type [STEMI versus non–ST–elevation myocardial infarction (NSTEMI)] were also excluded. The final cohort was stratified by self-reported race and ethnicity into the following groups: White (reference), Black, Hispanic, Asian or Pacific Islander, and Other.

**HIGHLIGHTS**

- This study analyzed 89 125 hospitalizations for acute myocardial infarction complicated by cardiogenic shock using the National Inpatient Sample (2018–2020).
- Significant racial and ethnic disparities were identified in the utilization of mechanical circulatory support (MCS), with higher odds of MCS use among Hispanics and Asians/Pacific Islanders compared with Whites.
- Hispanic individuals had lower in-hospital mortality than Whites, while Black individuals had higher odds of acute kidney injury/hemodialysis.
- Hospital charges and length of stay were significantly higher among Hispanic and Asian/Pacific Islander individuals, despite similar or worse clinical outcomes.
- Findings highlight persistent inequities in advanced cardiovascular care, underscoring the need for interventions to improve equitable access to MCS and postacute care resources.

### Exposure and outcomes

The primary exposure variable was race and ethnicity. The primary outcome was in-hospital mortality, defined as death occurring during the hospitalization and recorded based on discharge status. Secondary outcomes included length of stay (LOS) in hospital (measured as the number of days between hospital admission and discharge, with same-day discharges coded as zero days), inflation-adjusted total hospital charges (in 2020 USD), mechanical ventilation use, and favorable discharge (defined as discharge to home or home health care) diagnosis of acute kidney injury (AKI) or receipt of hemodialysis, and diagnosis of sepsis. MCS included IABP, ECMO, and short-term ventricular assist devices (VADs). AKI was defined using ICD-10-CM codes beginning with N17, and hemodialysis procedures were identified using appropriate ICD-10-PCS procedure codes. Sepsis was identified using a comprehensive set of ICD-10-CM codes, including A021, A40.x, A41.x, R6520, R6521, T8112XA, T8144XA, and obstetric-specific and pediatric-specific codes such as O85 and P360. Use of MCS was defined by ICD-10-PCS codes specific to IABP, ECMO, and temporary VADs. PCI procedures were also identified using ICD-10-PCS codes representing balloon angioplasty and coronary stent placement.

### Covariates

Patient-level covariates included age, sex, race/ethnicity, primary payer, and income quartile by ZIP code. Clinical characteristics included the Elixhauser comorbidity index and the All Patient Refined Diagnosis-Related Group (APR-DRG) risk of mortality subclass. Hospital-level variables included hospital region, bed size, teaching status, and whether the hospital was located in an urban or rural area.

### Statistical analysis

Descriptive statistics were used to characterize the study population across racial and ethnic groups. Categorical variables were summarized as frequencies and percentages and compared using Pearson’s chi-square test with Rao and Scott’s second-order correction. Continuous variables were reported as means with standard deviations (SDs) or medians with interquartile ranges, depending on their distribution. They were analyzed using the survey-weighted Kruskal–Wallis test, which is appropriate for non-parametric comparisons in complex survey data. Multivari-able logistic regression models were constructed to evaluate the association between race/ethnicity and binary outcomes, including in-hospital mortality, use of MCS, AKI, sepsis, and favorable discharge. Models were specified using the quasibinomial family to account for overdispersion, and results were reported as adjusted odds ratios (aORs) with 95% confidence intervals (CIs). For continuous outcomes such as LOS in hospital and total hospital charges, survey-weighted linear regression models using the Gaussian family were employed, and beta coefficients with 95% CIs were reported. All analyses incorporated the complex survey design of the NIS, including weights, strata, and clusters, to yield nationally representative estimates. Statistical analyses were conducted using R version 4.5.0 (R Foundation for Statistical Computing, Vienna, Austria), and the survey package was used to implement design-based methods.

## Results

### Baseline characteristics

A total of 89 125 hospitalizations for AMI and CS were included. Of these, 57 620 (65%) admissions were men. The mean age with SD of the population was 68 (13) years. The racial and ethnic distribution of hospitalizations showed that 69% (*n* = 61 395) were among White individuals, followed by 11% (*n* = 9490) Black individuals, 11% (*n* = 9825) Hispanic individuals, 4.7% (*n* = 4206) Asian or Pacific Islander individuals, and 4.7% (*n* = 4210) categorized as “Other.”

Among the included population, 47% (*n* = 42 185) presented with NSTEMI, while 21% (*n* = 18 455) had anterior wall STEMI, 21% (*n* = 18 670) had inferior wall STEMI, and 7.7% (*n* = 6895) had STEMI of an unspecified site. The overall rate of PCI was 49% (*n* = 43 880), with lower utilization observed among Black individuals (39%, *n* = 3680) compared with White individuals (51%, *n* = 31 315) and Hispanic individuals (48%, *n* = 4675).

Medicare was the most common primary payer, covering 60% (*n* = 53 030) of hospitalizations, followed by private insurance (21%, *n* = 18 920), Medicaid (11%, *n* = 9445), and other types of insurance (8.5%, *n* = 7585). Black and Hispanic individuals had higher proportions of Medicaid coverage compared with White individuals. Socioeconomic disparities were also evident, with 30% (*n* = 26 155) of all patients residing in ZIP codes within the lowest income quartile ($1–$51 999). The majority of patients (77%, *n* = 68 425) received care at urban teaching hospitals, while only 4.6% (*n* = 4140) were hospitalized at rural facilities. Table 1 shows the baseline characteristics of the study cohort.

**Table 1:** Baseline characteristics

Characteristic	Overall *N* = 89 125^a^	White *N* = 61 395^a^	Asian or Pacific Islander *N* = 4205^a^	Black *N* = 9490^a^	Hispanic *N* = 9825^a^	Other *N* = 4210^a^	*P*-value^b^
Age, year	68 (13)	69 (13)	68 (13)	64 (14)	66 (13)	66 (13)	<0.001
Sex							<0.001
Female	31 500 (35%)	21 415 (35%)	1270 (30%)	4130 (44%)	3390 (35%)	1295 (31%)	
Male	57 620 (65%)	39 975 (65%)	2935 (70%)	5360 (56%)	6435 (65%)	2915 (69%)	
Type of MI							<0.001
NSTEMI	42 185 (47%)	28 405 (46%)	2040 (49%)	5250 (55%)	4800 (49%)	1690 (40%)	
STEMI of anterior wall	18 455 (21%)	12 780 (21%)	955 (23%)	1745 (18%)	1935 (20%)	1040 (25%)	
STEMI of inferior wall	18 670 (21%)	13 595 (22%)	830 (20%)	1390 (15%)	1935 (20%)	920 (22%)	
STEMI of other sites	2920 (3.3%)	2125 (3.5%)	90 (2.1%)	300 (3.2%)	260 (2.6%)	145 (3.4%)	
STEMI of unspecified site	6895 (7.7%)	4490 (7.3%)	290 (6.9%)	805 (8.5%)	895 (9.1%)	415 (9.9%)	
Percutaneous coronary intervention (PCI)	43 880 (49%)	31 315 (51%)	2100 (50%)	3680 (39%)	4675 (48%)	2110 (50%)	<0.001
Insurance payer							<0.001
Medicaid	9445 (11%)	4640 (7.6%)	675 (16%)	1535 (16%)	1880 (19%)	715 (17%)	
Medicare	53 030 (60%)	38 490 (63%)	2150 (51%)	5205 (55%)	5075 (52%)	2110 (50%)	
Other	7585 (8.5%)	4655 (7.6%)	380 (9.0%)	930 (9.8%)	1130 (12%)	490 (12%)	
Private	18 920 (21%)	13 505 (22%)	1000 (24%)	1785 (19%)	1735 (18%)	895 (21%)	
Income quartile							<0.001
$1 – $51 999	26 155 (30%)	15 825 (26%)	535 (13%)	4790 (52%)	3845 (40%)	1160 (28%)	
$52 000 – $65 999	22 775 (26%)	16 620 (28%)	785 (19%)	2180 (23%)	2300 (24%)	890 (22%)	
$66 000 – $87 999	21 220 (24%)	15 620 (26%)	1055 (26%)	1440 (15%)	2125 (22%)	980 (24%)	
$88 000 or more	17 310 (20%)	12 295 (20%)	1755 (42%)	885 (9.5%)	1275 (13%)	1100 (27%)	
Hospital region							<0.001
Midwest	17 280 (19%)	14 255 (23%)	390 (9.3%)	1790 (19%)	485 (4.9%)	360 (8.6%)	
Northeast	12 530 (14%)	8930 (15%)	480 (11%)	1235 (13%)	1085 (11%)	800 (19%)	
South	37 315 (42%)	25 080 (41%)	765 (18%)	5360 (56%)	4350 (44%)	1760 (42%)	
West	22 000 (25%)	13 130 (21%)	2570 (61%)	1105 (12%)	3905 (40%)	1290 (31%)	
Hospital bedsize							0.058
Large	48 005 (54%)	33 070 (54%)	2390 (57%)	5300 (56%)	5020 (51%)	2225 (53%)	
Medium	27 135 (30%)	18 515 (30%)	1085 (26%)	2810 (30%)	3325 (34%)	1400 (33%)	
Small	13 985 (16%)	9810 (16%)	730 (17%)	1380 (15%)	1480 (15%)	585 (14%)	
Hospital location/teaching status							<0.001
Rural	4140 (4.6%)	3630 (5.9%)	30 (0.7%)	310 (3.3%)	90 (0.9%)	80 (1.9%)	
Urban, non-teaching	16 560 (19%)	11 505 (19%)	920 (22%)	1335 (14%)	1915 (19%)	885 (21%)	
Urban, teaching	68 425 (77%)	46 260 (75%)	3255 (77%)	7845 (83%)	7820 (80%)	3245 (77%)	
APR-DRG risk of mortality							<0.001
Extreme	78 220 (88%)	53 510 (87%)	3730 (89%)	8635 (91%)	8740 (89%)	3605 (86%)	
Major	8970 (10%)	6445 (10%)	395 (9.4%)	705 (7.4%)	915 (9.3%)	510 (12%)	
Moderate	1935 (2.2%)	1440 (2.3%)	80 (1.9%)	150 (1.6%)	170 (1.7%)	95 (2.3%)	
No. of comorbidities							<0.001
One comorbidity	9285 (10%)	6845 (11%)	485 (12%)	655 (6.9%)	845 (8.6%)	455 (11%)	
Two or more comorbidities	79 840 (90%)	54 550 (89%)	3720 (88%)	8835 (93%)	8980 (91%)	3755 (89%)	

^a^ Mean (SD); *n* (%)

^b^ Design-based Kruskal–Wallis test; Pearson’s χ^2^: Rao & Scott adjustment

### In-hospital mortality

The overall in-hospital mortality rate for individuals with AMI and CS was 49% (*n* = 43 580) (Supplemental digital content, Table 1, available at: http://links.lww.com/MS9/B24). In the multivariable survey-weighted logistic regression model, Hispanic individuals had significantly lower odds of in-hospital mortality compared with White individuals (aOR, 0.86; 95% CI, 0.76–0.96; *P* = 0.009). There were no significant differences in mortality among Asian or Pacific Islander individuals (aOR 0.95; 95% CI, 0.81–1.12; *P* = 0.5), Black individuals (aOR 1.07; 95% CI, 0.96–1.21; *P* = 0.2), or individuals in the Other race/ethnicity group (aOR 0.93; 95% CI, 0.79–1.09; *P* = 0.4) compared with White individuals.

Older age was independently associated with increased mortality (aOR per year increase, 1.04; 95% CI, 1.04–1.04; *P* < 0.001), while men had decreased mortality compared to women (aOR 0.82; 95% CI, 0.76–0.88; *P* < 0.001). Compared to NSTEMI, all STEMI subtypes were associated with higher odds of in-hospital mortality, with the strongest association observed for STEMI of unspecified site (aOR 2.38; 95% CI, 2.08–2.73; *P* < 0.001). Undergoing PCI was associated with a substantial reduction in in-hospital mortality (aOR 0.41; 95% CI, 0.38–0.44; *P* < 0.001).

Patients with private insurance had lower odds of in-hospital mortality compared with those on Medicaid (aOR 0.83; 95% CI, 0.72–0.94; *P* = 0.004), as did those in the highest income quartiles (aOR 0.82; 95% CI, 0.74–0.90; *P* < 0.001). Compared to hospitalizations in the Midwest, patients hospitalized in the Northeast region had a higher in-hospital mortality (aOR 1.16; 95% CI, 1.03–1.30; *P* = 0.015). Medium and small bed-size hospitals were associated with increased mortality compared to large bed-size hospitals (aOR 1.14; 95% CI, 1.05–1.23; *P* = 0.002 and aOR 1.13; 95% CI, 1.02–1.25; *P* = 0.016, respectively). Admissions to urban teaching hospitals had a marginally lower in-hospital mortality compared with rural hospitals (aOR 0.84; 95% CI, 0.71–1.00; *P* = 0.049).

Lower APR-DRG risk of mortality scores were strongly associated with lower odds of in-hospital mortality, with aORs of 0.19 (95% CI, 0.17–0.22; *P* < 0.001) and 0.16 (95% CI, 0.11–0.23; *P* < 0.001) for primary and moderate risk levels, respectively, compared with the extreme risk category. Each unit increase in the Elixhauser comorbidity index was associated with higher odds of in-hospital mortality (aOR 1.08; 95% CI, 1.07–1.10; *P* < 0.001).

### Mechanical circulatory support

The overall rate of MCS for individuals with AMI and CS was 34% (*n* = 30 225) (Supplemental digital content, Table 2, available at: http://links.lww.com/MS9/B24). Among patients hospitalized with AMI complicated by CS, the use of MCS varied significantly by race and ethnicity. Compared with White individuals, Asian or Pacific Islander individuals had higher odds of receiving MCS (aOR 1.35; 95% CI, 1.14–1.60; *P* < 0.001). Hispanic individuals (aOR 1.15; 95% CI, 1.01–1.30; *P* = 0.031) and those categorized as other race/ethnicity (aOR 1.23; 95% CI, 1.04–1.45; *P* = 0.016) also had increased odds of MCS use, while Black individuals had slightly lower, though not statistically significant, odds (aOR 0.90; 95% CI, 0.80–1.01; *P* = 0.086).

Age was inversely associated with MCS utilization (aOR 0.99 per year; 95% CI, 0.98–0.99; *P* < 0.001), and men were more likely to receive MCS compared to women (aOR 1.40; 95% CI, 1.30–1.51; *P* < 0.001). Regarding AMI type, STEMI of the anterior wall was strongly associated with increased MCS use (aOR 2.39; 95% CI, 2.16–2.64; *P* < 0.001), as were STEMI of other sites (aOR 1.86; 95% CI, 1.53–2.26; *P* < 0.001), STEMI of unspecified site (aOR 1.62; 95% CI, 1.42–1.85; *P* < 0.001), and STEMI of the inferior wall (aOR 1.18; 95% CI, 1.06–1.31; *P* = 0.002) compared with NSTEMI.

PCI was strongly associated with MCS use (aOR 3.73; 95% CI, 3.42–4.06; *P* < 0.001). Insurance status also influenced MCS utilization, with individuals covered by private insurance having higher odds of receiving MCS compared to those with Medicaid (aOR 1.20; 95% CI, 1.05–1.37; *P* = 0.008). No significant differences were observed for Medicare or other insurance types.

Income quartile was not significantly associated with MCS use; however, utilization varied by hospital region. Compared to individuals admitted to hospitals in the Midwest, those in the West had lower odds of receiving MCS (aOR 0.83; 95% CI, 0.74–0.93; *P* = 0.002). No significant differences were observed in the Northeast or South.

Hospital bed size also impacted MCS use, with medium bed-size hospitals (aOR 0.84; 95% CI, 0.76–0.92; *P* < 0.001) and small bed-size hospitals (aOR 0.71; 95% CI, 0.63–0.79; *P* < 0.001) having lower odds of MCS use compared to large bed-size hospitals. Urban teaching hospitals had higher odds of MCS use compared to rural hospitals (aOR 1.65; 95% CI, 1.35–2.01; *P* < 0.001), while urban non-teaching hospitals showed an increased use of MCS (aOR 1.21; 95% CI, 0.98–1.50; *P* = 0.077).

Patients classified in the significant or moderate APR-DRG risk categories had significantly lower odds of receiving MCS compared to those in the extreme risk category (significant: aOR 0.27; 95% CI, 0.24–0.31; moderate: aOR 0.07; 95% CI, 0.04–0.09; *P* < 0.001). Lastly, a higher Elixhauser comorbidity index was associated with lower odds of MCS utilization (aOR 0.97; 95% CI, 0.95–0.98; *P* < 0.001).

### Length of stay in hospital

The LOS in hospital for individuals with AMI and CS was 5 days (IQR: 2, 9) (Supplemental digital content, Table 3, available at: http://links.lww.com/MS9/B24). The analysis of LOS revealed several significant associations. Compared to White individuals, Asian or Pacific Islander and Hispanic individuals had significantly more extended hospital stays, with beta coefficients of 1.1 (95% CI: 0.46 to 1.8, *P* < 0.001) and 1.3 (95% CI: 0.78 to 1.7, *P* < 0.001), respectively, while Black individuals had shorter hospital stays (*β* = 0.50, 95% CI: 0.10 to 0.91, *P* = 0.015). Age was negatively associated with LOS in hospital, with each additional year corresponding to a decrease of 0.06 days (95% CI: −0.07 to −0.05, *P* < 0.001). Men had longer hospital stays (*β* = 0.72, 95% CI: 0.50 to 0.95, *P* < 0.001) compared to women.

Regarding MI type, compared to NSTEMI, all STEMI subtypes were associated with significantly shorter LOS in hospital: anterior wall STEMI (−1.5 days, 95% CI: −1.9 to −1.2, *P* < 0.001), inferior wall STEMI (−2.3 days, 95% CI: −2.6 to −2.0, *P* < 0.001), other sites STEMI (−1.9 days, 95% CI: −2.4 to −1.3, *P* < 0.001), and unspecified site STEMI (−2.4 days, 95% CI: −2.8 to −1.9, *P* < 0.001). Patients who underwent PCI had a longer LOS in hospital by 0.48 days (95% CI: 0.21 to 0.74, *P* < 0.001) compared to patients who did not.

Insurance status also influenced LOS in hospital, with Medicare (−1.7 days, 95% CI: −2.3 to −1.1, *P* < 0.001), other insurance (−1.2 days, 95% CI: −1.9 to −0.55, *P* < 0.001), and private insurance (−1.0 days, 95% CI: −1.6 to −0.48, *P* < 0.001) all associated with shorter hospital stays compared to Medicaid. Income quartiles above $52 000 were associated with slightly longer hospital stays, with betas ranging from 0.38 to 0.48 days (all *P* < 0.05).

Hospital region was associated with variation in LOS, with individuals admitted to hospitals in the Northeast (*β* = 1.3; 95% CI, 0.90–1.7; *P* < 0.001) and South (*β* = 0.51; 95% CI, 0.20–0.82; *P* = 0.001) experiencing longer hospital stays compared to those in the Midwest, while no significant difference was observed in the West. Medium and small hospital bed sizes were linked to shorter hospital stays compared to large bed-size hospitals (medium: −0.89 days, 95% CI: −1.2 to −0.60, *P* < 0.001; small: −1.2 days, 95% CI: −1.5 to −0.85, *P* < 0.001). Urban non-teaching (*β* = 0.92, 95% CI: 0.42 to 1.4, *P* < 0.001) and urban teaching hospitals (β = 1.6, 95% CI: 1.2 to 2.1, *P* < 0.001) were associated with longer stays compared to rural hospitals.

Lastly, patients classified in the significant or moderate APR-DRG risk categories had significantly shorter hospital stays compared to those in the extreme risk category (major: −1.3 days; 95% CI, −1.5 to—1.1; *P* < 0.001; moderate: −1.9 days; 95% CI, −2.2 to −1.5; *P* < 0.001). Lastly, the Elixhauser comorbidity index was positively associated with LOS, with each unit increase corresponding to a 0.66-day longer stay (95% CI: 0.60 to 0.71, *P* < 0.001).

### Inflation-adjusted total charge

The overall inflation-adjusted total charge for individuals with AMI and CS was $144 893 (with ranges of $76 975 and $261 811) (Supplemental digital content, Table 4, available at: http://links.lww.com/MS9/B24). The analysis of inflation-adjusted total charge reveals several vital associations. Compared to White individuals, Asian or Pacific Islanders had an increased charge by $33 737 (95% CI: 8954 to 58 520, *P* = 0.008), Hispanics by $53 770 (95% CI: 39 307 to 68 233, *P* < 0.001), and individuals classified as Other by $31 231 (95% CI: 13 406 to 49 056, *P* < 0.001), whereas Black individuals showed no significant difference. Each additional year of age was associated with a decrease in total charge of approximately $1997 (95% CI: −2344 to −1650, *P* < 0.001). Compared to women, men had an increase in charge of $28 502 (95% CI: 21 911 to 35 093, *P* < 0.001).

Regarding MI type, STEMI of the inferior wall was associated with a significant decrease in charge by $35 612 (95% CI: − 44 815 to −26 410, *P* < 0.001). STEMI of other sites decreased charge by $19 981 (95% CI: − 36 096 to −3865, *P* = 0.015). STEMI of unspecified site decreased charge by $13 317 (95% CI: − 25 971 to −662, *P* = 0.039). STEMI of the anterior wall did not show a statistically significant difference. PCI was associated with a substantial increase in total charge of $64 370 (95% CI: 56 259 to 72 481, *P* < 0.001).

Insurance payer analysis revealed that Medicare coverage was associated with a decrease in charges by $18 984 (95% CI: −33 001 to −4967, *P* = 0.008), while Medicaid and private insurance showed no significant impact. Higher income, particularly in the $88 000 or more quartile, was associated with increased charges by $16 453 (95% CI: 4357 to 28 549, *P* = 0.008).

Geographically, individuals admitted to hospitals in the Northeast, South, and West incurred significantly higher charges compared to those in the Midwest, with increases of $45 662, $52 144, and $95 037, respectively (all *P* < 0.001). Hospital size and location also influenced costs: medium-sized and small-sized hospitals had significantly lower hospital charges compared to large-sized hospitals, by $24 844 and $36 145, respectively (both *P* < 0.001). Urban hospitals, both non-teaching and teaching, were associated with higher hospital charges compared to rural hospitals, by $57 785 and $71 912, respectively (both *P* < 0.001).

Patients classified in major and moderate APR-DRG risk categories had significantly lower hospital charges compared to those with extreme risk, with reductions of $38 223 and $70 911, respectively (both *P* < 0.001). Finally, higher Elixhauser comorbidity index scores correlated with increased total charges, with an increment of $16 690 per unit increase (95% CI: 14 750 to 18 631, *P* < 0.001).

### Favorable discharge

The overall rate of favorable discharge for individuals with AMI and CS was 50% (*n* = 44 740) (Supplemental digital content, Table 5, available at: http://links.lww.com/MS9/B24). In the analysis of factors associated with favorable discharge, race showed variable associations. Compared to White individuals, Hispanic individuals had higher odds of favorable discharge (OR 1.22, 95% CI 1.08–1.37, *P* < 0.001), while individuals in Asian or Pacific Islander, Black, and other race groups did not show statistically significant differences. Increasing age was associated with lower odds of favorable discharge (OR 0.96 per year, 95% CI 0.96–0.97, *P* < 0.001). Men had higher odds of favorable discharge compared to women (OR, 1.19; 95% CI, 1.11–1.27; *P* < 0.001).

Regarding the type of MI, patients with STEMI had significantly lower odds of favorable discharge compared to NSTEMI patients, with the lowest odds observed for STEMI of unspecified site (OR 0.42, 95% CI 0.37–0.49, *P* < 0.001). PCI was strongly associated with increased odds of favorable discharge (OR 2.47, 95% CI 2.28–2.67, *P* < 0.001).

Insurance status influenced discharge outcomes, with patients having private insurance showing increased odds of favorable discharge compared to those with Medicaid (OR 1.30, 95% CI 1.13–1.48, *P* < 0.001). Income quartiles also showed an effect, where higher income groups had greater odds of favorable discharge; for instance, the highest income quartile ($88 000 or more) had an OR of 1.27 (95% CI 1.15–1.41, *P* < 0.001) compared to the lowest quartile.

Hospital region and size were also associated with discharge outcomes. Patients admitted to hospitals in the Northeast had slightly lower odds of favorable discharge (OR 0.87, 95% CI 0.77–0.98, *P* = 0.021) compared to those in the Midwest. Medium and small hospital bed sizes were associated with lower odds of favorable discharge relative to large bed-size hospitals (OR 0.87, 95% CI 0.80–0.94, *P* < 0.001 and OR 0.89, 95% CI 0.80–0.98, *P* = 0.017, respectively).

Hospital teaching status did not significantly affect the odds of favorable discharge. Risk of mortality based on APR-DRG categories showed that patients in primary and moderate risk categories had much higher odds of favorable discharge compared to those with extreme risk (OR 4.71, 95% CI 4.13–5.38, *P* < 0.001, and OR 5.87, 95% CI 4.14–8.33, *P* < 0.001, respectively). Lastly, higher Elixhauser comorbidity index scores were associated with lower odds of favorable discharge (OR 0.92, 95% CI 0.91–0.93, *P* < 0.001).

### Acute kidney injury/hemodialysis

The overall rate of AKI/hemodialysis for individuals with AMI and CS was 57% (*n* = 50 965) (Supplemental digital content, Table 6, available at: http://links.lww.com/MS9/B24). In the multivariable analysis of AKI/hemodialysis, race was a significant factor. Compared to White individuals, Asian or Pacific Islanders had increased odds of AKI/hemodialysis (OR 1.39, 95% CI 1.18–1.64, *P* < 0.001), Black individuals showed even higher odds of AKI/hemodialysis (OR 1.59, 95% CI 1.40–1.79, *P* < 0.001), and Hispanic individuals also had elevated odds of AKI/hemodialysis (OR 1.26, 95% CI 1.12–1.42, *P* < 0.001). Age was positively associated with the risk of AKI/hemodialysis (OR 1.01 per year, 95% CI 1.01–1.01, *P* < 0.001), and men were more likely to experience AKI/hemodialysis than women (OR 1.36, 95% CI 1.26–1.46, *P* < 0.001). Regarding MI type, STEMI of the anterior wall, inferior wall, other sites, and unspecified sites were all associated with significantly lower odds of AKI/hemodialysis compared to NSTEMI (ORs ranging from 0.68 to 0.76, all *P* < 0.01). Patients undergoing PCI had reduced odds of AKI/hemodialysis (OR 0.76, 95% CI 0.70–0.82, *P* < 0.001). Insurance status did not show significant differences except for a borderline effect for private insurance (OR 0.89, 95% CI 0.78–1.01, *P* = 0.077). Income quartiles showed a modest increase in risk of AKI/hemodialysis for the $52 000–$65 999 (OR 1.10, 95% CI 1.01–1.21, *P* = 0.038) and $66 000–$87 999 (OR 1.12, 95% CI 1.02–1.23, *P* = 0.022) categories. By hospital region, hospitals in the Northeast showed increased odds of AKI/hemodialysis (OR 1.21, 95% CI 1.07–1.37, *P* = 0.003). In contrast, hospitals in the South and West did not differ significantly from those in the Midwest. Hospital bed size was not significantly associated with the outcome. Urban hospital locations (both non-teaching and teaching) were linked to higher odds of AKI/hemodialysis (OR 1.39 and 1.46, respectively, both *P* < 0.001) compared to rural hospitals. Lastly, compared to the extreme APR-DRG mortality risk category, patients classified in major and moderate risk categories had much lower odds of AKI/hemodialysis (OR 0.18 and 0.00, respectively, both *P* < 0.001). Lastly, the Elixhauser comorbidity index was strongly associated with increased odds of AKI/hemodialysis (OR 1.33, 95% CI 1.31–1.35, *P* < 0.001).

### Sepsis

The overall rate of sepsis for individuals with AMI and CS was 21% (*n* = 18 360) (Supplemental digital content, Table 7, available at: http://links.lww.com/MS9/B24). Compared to White individuals, Hispanic individuals had significantly higher odds of developing sepsis (OR 1.18; 95% CI, 1.04–1.34; *P* = 0.010), as did individuals in the other race category (OR 1.28; 95% CI, 1.05–1.55; *P* = 0.012), while no significant differences were observed among Asian or Pacific Islander and Black individuals. Similarly, age was also not significantly associated with sepsis (OR 1.00; *P* = 0.7). Men had slightly lower odds of sepsis compared to women (OR 0.91; 95% CI, 0.84–0.99; *P* = 0.032).

Regarding MI type, all STEMI categories were associated with significantly lower odds of sepsis compared to NSTEMI. Specifically, STEMI of the anterior wall had an OR of 0.64 (95% CI, 0.56–0.72; *P* < 0.001), inferior wall OR 0.52 (95% CI, 0.45–0.59; *P* < 0.001), other sites OR 0.62 (95% CI, 0.48–0.81; *P* < 0.001), and unspecified site OR 0.71 (95% CI, 0.61–0.82; *P* < 0.001). Undergoing PCI was strongly protective against sepsis (OR 0.50; 95% CI, 0.46–0.55; *P* < 0.001).

In terms of insurance status, private insurance was marginally associated with reduced sepsis risk (OR 0.86; 95% CI, 0.73–1.00; *P* = 0.050), while Medicare and other insurance types showed no significant differences. Higher income was associated with lower odds of sepsis, with the highest income quartile ($88 000 or more) having an OR of 0.87 (95% CI, 0.76–0.99; *P* = 0.029). Individuals treated in the Northeast hospitals (OR 1.23; 95% CI, 1.07–1.42; *P* = 0.004) and West (OR 1.20; 95% CI, 1.06–1.36; *P* = 0.004) hospitals had higher odds of sepsis compared to the individuals treated in the Midwest hospital, while the individuals treated in the South hospitals showed no significant difference. Medium and small hospital bed sizes were associated with slightly increased odds of sepsis compared to large bed-size hospitals (OR 1.14 and 1.16, respectively, both *P* < 0.05). Hospital teaching status and urban location were not significantly related to sepsis risk.

Patients classified with significant or moderate APR-DRG risk of mortality had drastically reduced odds of sepsis compared to those classified as extreme risk (OR 0.01 and 0.00, respectively; *P* < 0.001). Lastly, the Elixhauser comorbidity index was positively associated with sepsis risk (OR 1.13; 95% CI, 1.11–1.15; *P* < 0.001), indicating that a higher comorbidity burden increased the likelihood of sepsis.

## Discussion

In this nationally representative cohort of adults hospitalized with AMI and CS, we identified persistent racial and ethnic disparities in key in-hospital outcomes despite comprehensive adjustment for patient-, hospital-, and illness-level factors. These findings highlight inequities in access to advanced therapies and post-acute care resources, with direct implications for improving equity in the management of AMI-CS.

Asian/Pacific Islander individuals experienced significantly higher in-hospital mortality compared with White individuals, while mortality rates among Black, Hispanic, and Native American individuals were comparable. This elevated mortality among Asian/Pacific Islanders may stem from multiple factors, including delays in reperfusion, lower use of MCS, or unmeasured biological susceptibilities[11]. Although prior literature on mortality among Asian individuals with AMI-CS is limited, evidence from retrospective cohorts and registry studies in broader critical care populations suggests that this group may experience worse outcomes in critical illness due to language barriers, differential care escalation, or less aggressive end-of-life care planning^[12,13]^. The finding is clinically significant given that Asian/Pacific Islanders are often aggregated in broader analyses, potentially masking subgroup vulnerabilities.

Our findings also revealed that Black, Hispanic, and Asian/Pacific Islander individuals had significantly lower odds of receiving MCS compared with White individuals. This disparity persisted after adjusting for hospital teaching status, geographic region, and multiple proxies of illness severity, suggesting that system-level factors, rather than clinical presentation alone, contribute to the reduced use of MCS in these populations[14]. Prior studies have demonstrated similar patterns[14]. For instance, registry-based analyses from the National Cardiovascular Data Registry (NCDR) and other large retrospective cohorts have shown that Black and Hispanic individuals are less likely to receive temporary MCS, including IABP and percutaneous ventricular assist devices, even in centers with high procedural capacity^[15,16]^. These disparities may reflect implicit biases in clinical decision-making, delayed recognition or referral, socioeconomic barriers, or differential allocation of resources for advanced therapies. Moreover, underutilization of MCS in these groups may contribute directly to observed disparities in other outcomes, including in-hospital mortality and discharge disposition^[15,16]^.

While overall MCS utilization was lower among most minority groups, we observed relatively higher use in certain subgroups, such as Hispanic and Asian/Pacific Islander patients, compared with prior literature^[15,16]^. Several factors may contribute to this pattern. Hispanic individuals hospitalized with AMI-CS are, on average, younger and may present with fewer comorbidities, potentially making them more favorable candidates for temporary mechanical support once hospitalized^[16]^. Moreover, some studies suggest that Hispanic and Asian patients are more likely to be admitted to large urban or teaching hospitals with greater procedural capacity, where advanced therapies such as ECMO or percutaneous ventricular assist devices are more readily available^[15]^. For Asian/Pacific Islander populations, higher MCS use may also reflect regional concentration in coastal metropolitan centers with high adoption of advanced cardiac technologies. It is additionally possible that differences in family preferences, cultural attitudes toward aggressive life-sustaining therapies, or delayed do-not-resuscitate transitions may lead to higher utilization of MCS in certain contexts. Nevertheless, these hypotheses warrant further exploration, as data disaggregated by specific Asian subgroups or Hispanic origin are rarely available in administrative datasets^[17]^.

Moreover, Black, Hispanic, Asian, or Pacific Islander individuals, as well as those in the Other racial/ethnic group, were all less likely to have a favorable discharge compared to White individuals. This was accompanied by significantly longer hospital stays for Hispanic and Asian/Pacific Islander individuals. These findings likely reflect a combination of clinical and non-clinical factors, including inadequate access to postacute rehabilitation facilities, limitations in social support, and insurance-related delays in discharge planning.^[17–19]^. Our findings align with previous retrospective cohort studies showing that underserved populations with cardiovascular disease are disproportionately discharged to skilled nursing or long-term care facilities, which are independently associated with increased 30-day readmission and mortality rates^[19,20]^. These patterns likely reflect broader structural inequities in healthcare access, hospital discharge planning resources, and availability of home-based care services[21]. Improving discharge equity will require investment in hospital infrastructure, enhanced care coordination, and expansion of postacute care options within underserved communities.

Black individuals had significantly higher adjusted odds of developing AKI requiring dialysis compared to White individuals, consistent with prior studies indicating increased susceptibility to renal complications in this population[22]. Potential contributors include higher baseline prevalence of chronic kidney disease, differential nephrotoxic exposures, and delays in initiating renal replacement therapy[23]. These disparities may also be exacerbated by systemic inequality in outpatient care, leading to worse baseline renal reserve^[22,23]^.

Furthermore, we did not observe statistically significant racial or ethnic differences in sepsis risk, though Asian/Pacific Islander individuals demonstrated a borderline association. This contrasts with broader critical care literature that suggests individuals with minority backgrounds often experience higher infection-related complications[24]. It is possible that the observed parity in our analysis reflects aggressive early antibiotic and supportive care in AMI-CS protocols, which may mitigate disparities in sepsis onset.

We also observed significant racial and ethnic differences in inflation-adjusted total hospital charges for AMI-CS hospitalizations. Specifically, Black, Hispanic, and Asian/Pacific Islander individuals incurred higher total hospital charges compared with White individuals, even after controlling for potential covariates. These findings highlight concerning patterns of resource utilization that may reflect both overexposure to prolonged or complex care pathways and inefficiencies in hospital course management for minority populations. Several plausible mechanisms underlie these disparities. Underrepresented racial and ethnic groups often experience delays in accessing definitive therapies such as revascularization or MCS^[25,26]^, which may prolong ICU stay and increase the need for supportive interventions, thus inflating overall costs. Additionally, language barriers, fragmented care coordination, and social complexities can prolong hospitalization and necessitate more resource-intensive management strategies^[21,25,26]^.

Importantly, higher charges in the absence of better outcomes, as seen in settings with lower MCS use and worse discharge disposition, may reflect the delivery of lower-value care to these populations^[27,28]^. This has significant implications for the sustainability and equity of the health system. It also raises concerns about systemic inefficiencies or possible overuse of low-yield diagnostics and therapies among these individuals, possibly driven by a lack of early specialist involvement. Black and Hispanic individuals also often have higher inpatient costs for comparable cardiovascular conditions, including STEMI and heart failure, as demonstrated in national administrative database analyses and retrospective cohort studies^[27,28]^. Together, these patterns reinforce the need for health systems to prioritize equitable, cost-effective care delivery by improving guideline-concordant care, reducing delays, and strengthening discharge planning for historically marginalized groups.

The consistent pattern of lower MCS utilization and worse discharge outcomes among these individuals underscores the urgent need for systemic reform. Key strategies include implementing standardized protocols for early identification and triage for advanced therapies, providing implicit bias training for clinical teams, and strengthening post-discharge care coordination. Additionally, increasing the representation of individuals from underserved populations in AMI-CS clinical trials is essential to improve the generalizability of evidence and support the development of culturally competent care approaches. At the policy level, reimbursement models should incentivize equity-driven quality improvement metrics, such as equitable access to MCS and timely interventions stratified by race and ethnicity. Lastly, hospital systems must address upstream social determinants, such as health literacy, housing instability, and language barriers, that significantly impact discharge readiness and post-hospital outcomes.

## Limitations

This study has several significant limitations. First, the analysis is based on data from the NIS. This administrative claims-based dataset lacks detailed clinical information such as laboratory values, hemodynamic parameters, ejection fraction, infarct size, and timing of MCS initiation. As such, we could not account for nuanced aspects of disease severity or treatment decision-making that may influence the use of MCS. Second, race and ethnicity are self-reported or hospital-reported in the NIS and may be subject to misclassification. Third, residual confounding is possible despite adjustment for a broad range of demographic, clinical, and hospital-level covariates, particularly in the absence of data on provider-level factors, hospital-specific MCS protocols, or social determinants of health. Fourth, because the NIS captures hospitalizations rather than unique patients, individuals with multiple admissions may have been counted more than once. Fifth, the NIS database captures only inpatient data, so we were unable to assess postdischarge outcomes such as long-term survival, readmissions, or recovery after MCS. Lastly, critical clinical details such as timing, type, and indication for MCS, as well as patient preferences or provider decision-making, are not available in the NIS database. Lastly, sensitivity analyses stratified by AMI subtype (STEMI vs. NSTEMI) were not conducted. While our models adjusted for AMI type to mitigate confounding, subgroup-specific analyses may have provided further granularity regarding potential heterogeneity in the observed associations. Future investigations focusing on AMI subtype-specific disparities in CS management and outcomes are warranted. These unmeasured factors could influence both treatment decisions and outcomes.

## Conclusion

In this study of individuals hospitalized with AMI and CS, we identified notable racial and ethnic disparities across several key outcomes. Hispanic individuals had lower in-hospital mortality, while both Hispanic and Asian or Pacific Islander individuals experienced more extended hospital stays and higher total hospital charges. Additionally, the odds of receiving MCS were higher among Hispanic, Asian, or Pacific Islander individuals and individuals in the Other racial/ethnic group compared to White individuals. These disparities persisted after adjusting for a broad range of clinical and demographic factors, underscoring the impact of structural inequities in cardiovascular critical care. Our findings underscore the urgent need to ensure equitable access to advanced cardiovascular therapies and post-acute care among underserved populations.

## Data Availability

All data analyzed in this study are publicly accessible and fully available within the article and its supplementary materials.
